# Copper Nanoparticles Decorated Alginate/Cobalt-Doped Cerium Oxide Composite Beads for Catalytic Reduction and Photodegradation of Organic Dyes

**DOI:** 10.3390/polym14204458

**Published:** 2022-10-21

**Authors:** Hamed A. Alshaikhi, Abdullah M. Asiri, Khalid A. Alamry, Hadi M. Marwani, Soliman Y. Alfifi, Sher Bahadar Khan

**Affiliations:** 1 Chemistry Department, Faculty of Science, King Abdulaziz University, P.O. Box 80203, Jeddah 21589, Saudi Arabia; 2 Center of Excellence for Advanced Materials Research, King Abdulaziz University, P.O. Box 80203, Jeddah 21589, Saudi Arabia

**Keywords:** copper nanoparticles, alginate, Co–CeO_2_, nanocatalyst, photodegradation, reduction, dyes, organic pollutants, water treatment

## Abstract

Cobalt-doped cerium oxide (Co–CeO_2_) was synthesized and wrapped inside alginate (Alg) hydrogel beads (Alg/Co–CeO_2_). Further, copper nanoparticles (Cu) were grown on Alg/Co–CeO_2_ beads. Cu decorated Alg/Co–CeO_2_ composite beads (Cu@Alg/Co–CeO_2_) were tested as a catalyst for the solar-assisted photodegradation and NaBH_4_-assisted reduction of organic pollutants. Among different dyes, Cu@Alg/Co–CeO_2_ was found to be the best catalyst for the photodegradation of acridine orange (ArO) under solar light and efficient in reducing methyl orange (MO) with the aid of NaBH_4_. Cu@Alg/Co–CeO_2_ decolorized ArO up to 75% in 5 h under solar light, while 97% of MO was reduced in 11 min. The decolorization efficiency of Cu@Alg/Co–CeO_2_ was further optimized by varying different parameters. Thus, the designed catalyst provides a promising way for efficient oxidation and reduction of pollutants from industrial effluents.

## 1. Introduction

Advances in the food automation, textile, paper, and leather industries have promoted the usage of organic dyes. These industries and tanneries are the main sources of organic pollutants, and their physical presence in wastewater has given the earth an unpleasant look, becoming a global concern [1,2,3]. The presence of these dyes’ pollutants at very low concentrations in wastewater cannot be removed by sedimentation and ordinary chemical degradation since dyes are very stable, carcinogenic, and mutagenic in nature for humans and living organisms [4,5]. Therefore, it is important to remove these effluents from discharged wastewater. Several methods have been used for their removal, such as biodegradation [6,7,8], thermal radiation [9], fenton [10,11], chemical reduction [12], microbial catabolism [13,14], ultrasonic excitement [15], and photocatalytic degradation [16,17,18]. Some of these methods cannot completely remove these effluents. Therefore, a green procedure, i.e., photocatalytic degradation and catalytic reduction, is needed to eliminate these hazardous pollutants. These methods require an efficient catalyst and sunlight/strong reducing agent to catalyze the degradation/reduction [16,17,18,19].

Metal oxides (MOs) and zero-valent metal nanoparticles (MNPs) have attracted significant attention because MOs have shown significant properties and have been used in different applications [16,17,18,19,20,21]. Therefore, fabricating a suitable catalyst possessing appreciable activity with fast electron donor and acceptor ability is highly required. Doped MOs and MNPs, i.e., Cu, Ag, Ni, Co, etc., have played a vital role in photocatalysis and catalytic reduction of organic pollutants [21,22,23]. However, these NPs are prone to aggregation due to their high surface energy, which limits their catalytic property [24]. Additionally, they cannot be easily separated from aqueous solution for reuse. Hence, various supportive surfaces have been employed to stabilize these MNPs [25,26,27,28,29].

Polymer composites and hydrogels combined with nanoparticles, which reinforce the mechanical properties of hydrogels, manifest more efficiently. They exhibit stimuli-responsive characteristics, including catalysis, drug degradation, elimination of aquatic pollution, and other features, which are reasons to consider them “smart” materials [30,31,32,33]. Concerning wastewater processing, hydrogel-based composites exhibit top efficacy in the reduction of various species for different contaminants [34]. Hydrogels being highly hydrophilic provides quasi-homogeneous traits to nanoparticles, hence improving their catalytic activity [35]. For more than a decade, a wide range of research has been carried out to improve incoming photocatalytic nanohybrids as emerging materials for wastewater remediation. A synthetic route may affect the catalytic performance of hybrid materials [36]. Metal-based nanomaterials have been given significant attention in different applications, especially their high efficiency in catalysis [37,38,39,40,41]. In a previous study, a metal oxide nanocatalyst was found to be highly selective, as it showed greater effectiveness in reducing potassium hexacyanoferrate (K_3_[Fe(CN)_6_]) [42]. Currently, new treatment technologies for dyes are needed, which can clear dyes from wastewater and minimize the exposure of toxic chemicals to humans and the environmental system [43]. Photocatalytic degradation and catalytic reduction are the most studied methods for dye removal and conversion of dyes into less toxic products [44].

Doped metal oxides have become one of the most effective materials used as catalysts, especially in nanosized materials. Such materials have shown exceptional characteristics in several applications, especially in reducing and degrading water pollutants. Doping by metals participates in regulating the electronic and catalytic properties of the catalyst and elevating surface area, thus enhancing its catalytic characteristics [35,42].

In this study, Co–CeO_2_ was simply prepared and then entrapped inside alginate hydrogel beads. Alg/Co–CeO_2_ beads were further dipped in copper solution, where the beads adsorbed Cu from the solution and were converted into Cu nanoparticles by treatment with NaBH_4_. Cu@Alg/Co–CeO_2_ was evaluated as a catalyst for the solar-assisted photodegradation and NaBH_4_-assisted reduction of organic pollutants. Cu@Alg/Co–CeO_2_ was found to be the best catalyst for the photodegradation of acridine orange (ArO) under solar light, as well as a competent catalyst for reducing MO with the aid of NaBH_4_.

## 2. Experimental Section

### 2.1. Chemicals and Reagents

Sodium alginate, cerium nitrate, cobalt nitrate, copper nitrate, 4-nitrophenol (4-NP), 2,6-dinitrophenol (2,6-DNP), sodium hydroxide, 2-nitrophenol (2-NP), acridine orange (ArO), methyl orange (MO), congo red (CR), methylene blue (MB), aluminum chloride, sodium borohydride, and all other utilized chemicals and solvents were purchased from Sigma Aldrich and BDH. Distilled water was utilized in all experiments.

### 2.2. Synthesis of Co–CeO_2_ Nanoparticles

To prepare the Co–CeO_2_ nanocomposite, 0.1 molar solution was prepared by dissolving 4.36 g of cerium nitrate and 5.83 g of cobalt nitrate in 100 mL of deionized water. NaOH was then added to increase the pH of the salt solution and then kept on heating (60 °C) with stirring. After 12 h, the precipitate was washed several times with distilled water, dried in an oven at 50 °C, and then calcined at 500 °C [45,46,47,48].

### 2.3. Preparation of Cu@Alg/Co–CeO_2_

To prepare Cu@Alg/Co–CeO_2_, Co–CeO_2_ was ground until it became powder, and then 2.0338 g of Co–CeO_2_ powder was taken and dispersed in 30 mL of Alg solution. The solution was mixed together by stirring. The mixture of Co–CeO_2_ with Alg was taken in a syringe and added dropwise from the mixture of Alg/Co–CeO_2_ to AlCl_3_ solution. Thus, a granular form of Alg/Co–CeO_2_ was obtained and left in the solution (AlCl_3_) for a while. Then, the granules were washed with distilled water and completely dried. Alg/Co–CeO_2_ granules were placed in the copper solution overnight. Alg/Co–CeO_2_ beads entrapped Cu ions and then treated with NaBH_4_ solution and converted into nanoparticles [49,50].

### 2.4. Apparatus

For morphology characterization of CeO_2_–Co_2_O_3_ and Cu@Alg/Co–CeO_2_, a scanning electron microscope was used, while for compositional analysis, an energy-dispersive spectrometer (EDS) was utilized. The morphology and particle size of the samples were studied using a scanning electron microscope (SEM: JSM-5910, JEOL). For this purpose, a small amount of the powder samples was stuck on aluminum stubs with the help of carbon conducting tape. The stubs were placed in an autofine coater (JFC-1600, JEOL) for sputtering with a thin layer of gold for 30 s. The stubs containing the samples were then placed in the sample chamber of the SEM. After evacuating the machine according to the standard procedures, the samples were investigated for their morphology. The distance of the sample from the tip of the electron gun and the accelerating voltage were adjusted to 10 mm and 15 kV, respectively. The same samples were used for EDX analysis. Removal of organic pollutants was observed by a UV–vis spectrophotometer (Thermo Scientific Evolution 300 UV–visible spectrophotometer, Waltham, MA, USA), which recorded the catalytic experiments at wavelengths between 200 and 800 nm.

### 2.5. Catalytic Reduction

Cu@Alg/Co–CeO_2_ was applied for the reduction of organic pollutants using NaBH_4_ as a reducing agent, and we evaluated its catalytic activity. Initially, 2.5 mL of pollutants (4-NP (0.13 mM), ArO (0.07 mM), CR (0.07 mM), MO (0.07 mM), MB (0.07 mM), 2,6-DNP (0.13 mM), 2-NP (0.13 mM), and K_3_[Fe(CN)_6_] (0.5 mM)) was mixed with 0.5 mL of NaBH_4_ (0.1 M) in a UV cuvette. The different amount (2–10 beads) of Cu@Alg/Co–CeO_2_ was introduced to the mixture, and the UV–vis spectrum was taken at different times. The effect of different parameters such as catalyst amount, reducing agent amount, and reusability was checked to optimize the method toward reduction of the most selective dye based on the study. The effectiveness of Cu@Alg/Co–CeO_2_ was assessed by Equation (1) [42]:

(1) $$\%\;\mathrm{Reduction}/\mathrm{Degradation}=\frac{C_{o-}C_{t}}{C_{o}}\times 100$$

where *C_o_* is the initial concentration/absorbance, and *C_t_* is the concentration/absorbance at time (*t*) of each individual pollutant.

### 2.6. Photocatalytic Degradation

Cu@Alg/Co–CeO_2_ was also tested as a photocatalyst for the degradation of MO, ArO, CR, and MB. Initially, 10 mL of each pollutant (MO (0.07 mM), ArO (0.07 mM), CR (0.07 mM) and MB (0.07 mM)) was taken in a beaker individually. Then, Cu@Alg/Co–CeO_2_ (4–10 beads) was mixed with the pollutant solution and kept under solar light. An amount of 3 mL of solution was taken from the reaction beaker at different times and then recording the UV–vis spectrum. The influence of different parameters such as photocatalyst amount, light source, and reusability was also investigated toward degradation of the most selective dye based on the study. The effectiveness of Cu@Alg/Co–CeO_2_ was assessed by Equation (1).

## 3. Results and Discussion

### 3.1. Characterization of Co–CeO_2_, Alg/Co–CeO_2_, and Cu@Alg/Co–CeO_2_

**Scheme 1:**
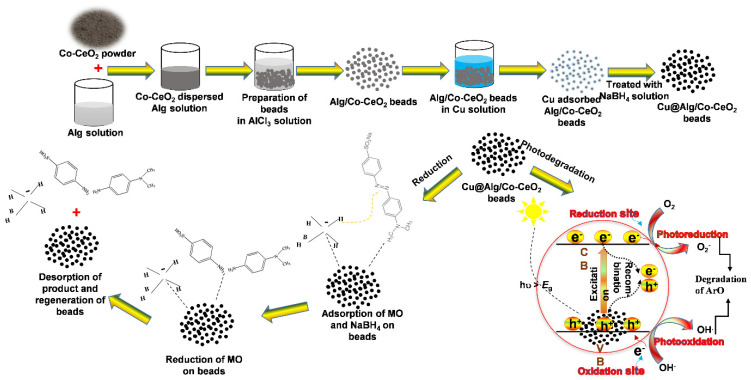
Schematic representation of preparation and catalytic properties of Cu@Alg/Co–CeO_2_.

First, Co–CeO_2_ was prepared and then dispersed in Alg solution. The mixed solution was crosslinked by AlCl_3_ and produced Alg/Co–CeO_2_ beads. Alg/Co–CeO_2_ beads were dipped in Cu salt solution, where Cu ions bonded with the COO–, OH, and O groups of Alg/Co–CeO_2_. The adsorbed Cu ions were converted into Cu nanoparticles by NaBH_4_, and thus Cu nanoparticles were grown inside and on the Alg/Co–CeO_2_ beads’ surface. The growth of Cu nanoparticles is shown in Scheme 1, and the reduction of Cu nanoparticles by NaBH_4_ is presented as follows:

2Cu^2+^ + 4BH_4_^−^ + 12H_2_O ⇒
2Cu^0^ + 4B(OH)_3_ + 14H_2_

**Figure 1:**
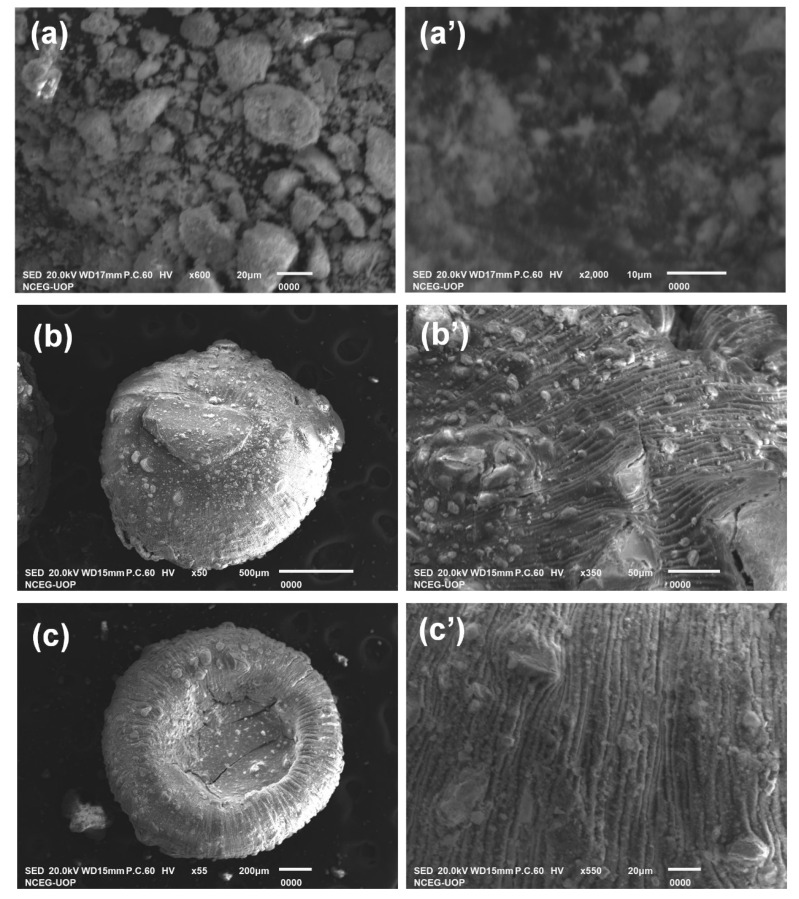
SEM images of (**a**,**a’**) Co–CeO_2_, (**b**,**b’**) Alg/Co–CeO_2_, and (**c**,**c’**) Cu@Alg/Co–CeO_2_.

The morphology of Co–CeO_2,_ Alg/Co–CeO_2_, and Cu@Alg/Co–CeO_2_ was assessed from SEM images. SEM images of Co–CeO_2,_ Alg/Co–CeO_2_, and Cu@Alg/Co–CeO_2_ are illustrated in Figure 1, where it can be clearly noticed that Co–CeO_2_ is grown in high quantities in the form of nanoparticles. Some parts of the picture show the accumulation and aggregation of nanoparticles in the case of Co–CeO_2_ (Figure 1a,a’). Alg/Co–CeO_2_ shows a loose-fitting rough and irregular surface. Alg/Co–CeO_2_ images show a rough surface with pores and grooves (Figure 1b,b’). Alg/Co–CeO_2_ images show well-dispersed particles in the hydrogel beads’ matrix. This suggests that the hydrogel beads have well-dispersed Co–CeO_2_ encapsulated inside. However, Cu@Alg/Co–CeO_2_ has a more compact morphology (Figure 1c,c’), along with well-dispersed particles in the hydrogel beads’ matrix. These particles reflect the presence of Cu nanoparticles along with well-dispersed Co–CeO_2_ inside the hydrogel beads. The morphology of Cu@Alg/Co–CeO_2_ changed to a more compact surface containing wrinkles after the growth of Cu nanoparticles on the surface. This indicates that copper ion adsorption, and further its conversion to Cu nanoparticles, causes shrinkage of the polymeric matrix and provides a more compact structure [51]. The growth of Cu nanoparticles causes a change in the surface morphology of Alg/Co–CeO_2_ hydrogels.

**Figure 2:**
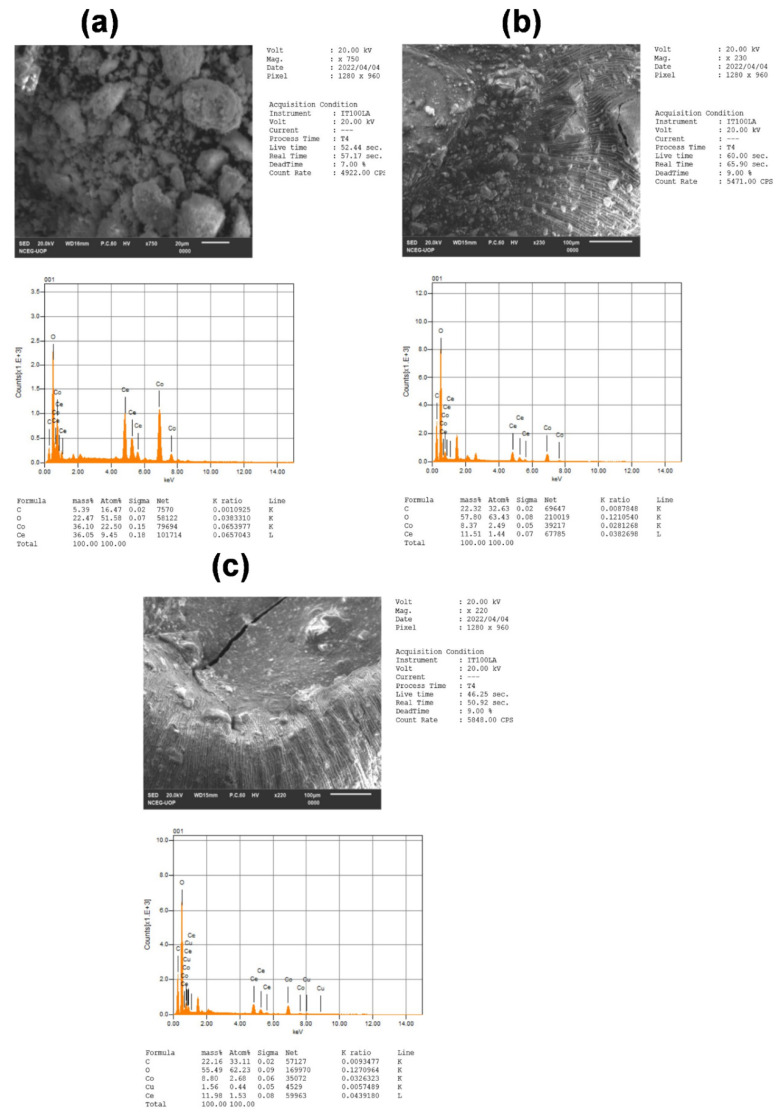
EDS spectra of (**a**) Co–CeO_2_, (**b**) Alg/Co–CeO_2_, and (**c**) Cu@Alg/Co–CeO_2_.

EDS was utilized to assure the elemental composition of Co–CeO_2,_ Alg/Co–CeO_2_, and Cu@Alg/Co–CeO_2_. The spectrum of Co–CeO_2_ exhibited Co (36.1 mass %), Ce (36.05 mass %), and O (22.47 mass %) peaks (Figure 2a), while the Alg/Co–CeO_2_ spectrum displayed Co, Ce, and O peaks along with C and O elements. Thus, the observed spectrum of Co–CeO_2_ confirms that Co–CeO_2_ composed of Co, Ce, and O elements, while Alg/Co–CeO_2_ contains both Co–CeO_2_ and Alg. C and O peaks reflect Alg, which is the major component of Alg/Co–CeO_2_ because the mass % of C and O is 22.32 and 57.80, while Co and Ce have a mass % of 8.80 and 11.51 (Figure 2b). EDS of Cu@Alg/Co–CeO_2_ exhibited peaks for C, O, Ce, Co, and Cu. C and O peaks represented Alg, Ce and Co due to Co–CeO_2_, while Cu appeared due to the presence of Cu nanoparticles (Figure 2c). The mass % obtained for C, O, Ce, Co, and Cu was 22.16, 55.49, 8.80, 11.89, and 1.56, respectively. EDS spectra also suggest that Co–CeO_2,_ Alg/Co–CeO_2_, and Cu@Alg/Co–CeO_2_ are pure because only Co, Ce, and O were observed in Co–CeO_2_, C, O, Co, and Ce in the case of Alg/Co–CeO_2_ and C, O, Co, Ce, and Cu in the case of Cu@Alg/Co–CeO_2_.

### 3.2. Water Purification Applications

Recently, several metal oxides have been utilized as catalysts for the removal of toxic pollutants owing to their superior catalytic activity during oxidation or reduction. Such processes are initiated by solar/UV light or strong reducing agents such as NaBH_4_ using metal oxide nanoparticles as catalysts. In the literature, different pollutants have been catalytically degraded/reduced by employing diverse metallic oxides with the aid of light or NaBH_4_ [52,53,54]. Therefore, Cu@Alg/Co–CeO_2_ was tested for the catalytic removal of dyes and other contaminants.

#### 3.2.1. Catalytic Reduction

Cu@Alg/Co–CeO_2_ demonstrated superior catalytic performance in the presence of NaBH_4_. Pure Co–CeO_2_ exhibited good activity, but the leaching and reusability of Co–CeO_2_ in powder form is a challenging task. As it is extremely difficult to separate Co–CeO_2_ in nanosized powder form from the reaction mixture for reuse, as well as leaching via filtration, Co–CeO_2_ was embedded inside the Alg beads to control the leaching of Co–CeO_2_ and increase the possibility of reusability. The catalyst in bead form can easily be pulled from the reaction mixture, washed, and reused in another catalytic reaction. Co–CeO_2_ was wrapped inside Alg beads by dispersing it first in Alg solution and then crosslinking it with AlCl_3_. The beads were further subjected to the growth of Cu nanoparticles, and the developed Cu@Alg/Co–CeO_2_ catalyst was tested in reducing different organic pollutants such as dyes, K_3_[Fe(CN)_6_] and nitrophenols with the aid of NaBH_4_. Different pollutants such as 4-NP, ArO, CR, MO, MB, 2,6-DNP, 2-NP, and K_3_[Fe(CN)_6_] were chosen for the current study, where Cu@Alg/Co–CeO_2_ was used as a catalyst with the aid of NaBH_4_ to evaluate its catalytic activity and efficacy as a nanocatalyst. Cu@Alg/Co–CeO_2_ was added to each individual pollutant along with NaBH_4_.

**Figure 3:**
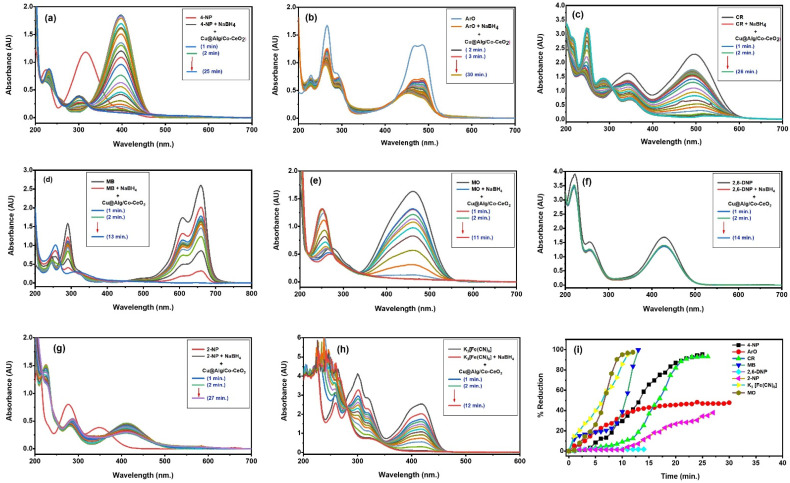
UV–vis absorption spectra for the reduction of pollutants: (**a**) 4-NP (0.13 mM), (**b**) ArO (0.07 mM), (**c**) CR (0.07 mM), (**d**) MO (0.07 mM), (**e**) MB (0.07 mM), (**f**) 2,6-DNP (0.13 mM), (**g**) 2-NP (0.13 mM), and (**h**) K_3_[Fe(CN)_6_] (0.5 mM)] using 0.5 mL NaBH_4_ (0.1 M) and Cu@Alg/Co–CeO_2_ catalyst (4 beads). (**i**) % Reduction of pollutants as a function of time.

The study steps were as follows: Initially, a 2.5 mL (4-NP (0.13 mM), ArO (0.07 mM), CR (0.07 mM), MO (0.07 mM), MB (0.07 mM), 2,6-DNP (0.13 mM), 2-NP (0.13 mM), and K_3_[Fe(CN)_6_] (0.5 mM)) of each individual pollutant was taken, and UV–vis absorption was measured for the pure pollutant. After that, 0.5 mL of the NaBH_4_ (concentration, 0.1 mM) reducing agent was mixed with the pollutant, and UV–vis absorption was measured for each pollutant with the aid of the reducing agent NaBH_4_; then, four beads of Cu@Alg/Co–CeO_2_ were introduced to the mixture, and the UV–vis spectrum of the mixture was taken every minute until the pollutant was completely reduced. Figure 3 displays that the absorbance band of the pollutants decreased steadily when completing the reduction reaction. These findings support that the pollutants were reduced, forming less toxic products by transmitting the electron donor BH_4_^−^ to the catalyst and moving electrons to the acceptor pollutant molecules.

**Figure 4:**
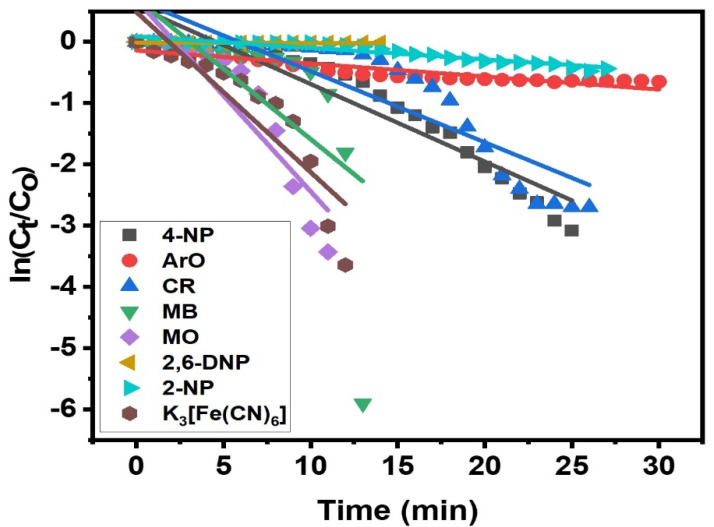
Kinetic study for reduction of 4-NP (0.13 mM), ArO (0.07 mM), CR (0.07 mM), MO (0.07 mM), MB (0.07 mM), 2,6-DNP (0.13 mM), 2-NP (0.13 mM), and K_3_[Fe(CN)_6_] (0.5 mM) using 0.5 mL NaBH_4_ (0.1 M) and Cu@Alg/Co–CeO_2_ catalyst (4 beads).

The % reduction was calculated based on Equation (1), and the reduction rates of 4-NP (0.13 mM), ArO (0.07 mM), CR (0.07 mM), MO (0.07 mM), MB (0.07 mM), 2,6-DNP (0.13 mM), 2-NP (0.13 mM), and K_3_[Fe(CN)_6_] (0.5 mM) with the aid of NaBH_4_ were calculated based on Equation (2):

ln *C_t_*/*C_o_* = ln *A_t_*/*A_o_* = −*K*t (2)

where *C_t_* and *C_o_* are pollutant concentrations, in which A*_t_* (absorbance at a specific time) and A*_o_* (initial absorbance) are equal to C*_t_* and C*_o_*, respectively. *k_app_* can be acquired from plotting ln(*C_t_*/*C_o_*) vs. reduction time (t). ln(*A_t_*/*A_0_*) vs. time (t) and kinetics of MO reduction by Cu@Alg/Co–CeO_2_ are plotted in Figure 4.

The % reduction of the selected pollutants using Cu@Alg/Co–CeO_2_ catalyst was found to be high (>95%) for 4-NP, CR, MB, MO, and K_3_[Fe(CN)_6_] and low (<40%) for ArO, 2,6-DNP, and 2-NP. Table 1 clearly displays % reduction, consumed time, and rate constant for the reduction of each compound. It is clear that MO was reduced in the shortest time compared with the other studied pollutants, providing the highest rate constant equal to 0.3129 min^−1^ using the Cu@Alg/Co–CeO_2_ catalyst.

Cu@Alg/Co–CeO_2_ demonstrated superior catalytic activity toward MO in the presence of NaBH_4_. Thus, Cu@Alg/Co–CeO_2_ was more effective for MO reduction. So, MO was selected for detailed investigation, where the optimization of catalyst amount, NaBH_4_ concentration, and recyclability was performed.

MO reduction by NaBH_4_ was conducted by employing Cu@Alg/Co–CeO_2_ beads as a catalyst. First, Cu@Alg/Co–CeO_2_ was introduced into the MO solution along with NaBH_4_. A decrease in MO absorbance peaks at 460 nm and 270 nm was noticed until the termination of the reaction, with a new peak appearing at 247 nm. Such findings reveal that the azo group (–N=N–) in MO was reduced, producing new products by the transmitting electron donor BH_4_^-^ to the nanocatalyst beads, thus handing over electrons to the acceptor MO molecules. The –N=N– bonds broke down to –N–N– bonds, which decolorized the MO solution [50].

**Figure 5:**
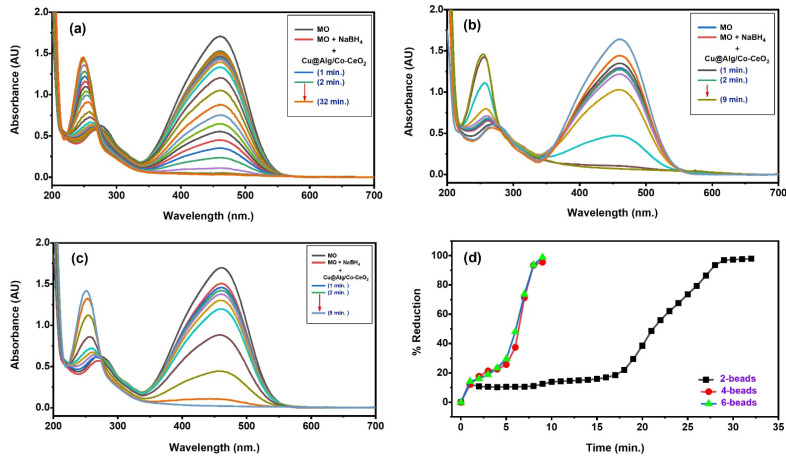
UV–vis absorption spectra for the reduction of MO (0.07 mM) using 0.25 mL NaBH_4_ (0.1 M) and (**a**) 2 beads, (**b**) 4 beads, and (**c**) 6 beads of Cu@Alg/Co–CeO_2_ catalyst. (**d**) % Reduction of MO as a function of time.

Further, we studied the influence of Cu@Alg/Co–CeO_2_ amount on MO catalytic reduction with the aid of NaBH_4_, and therefore, the effect of Cu@Alg/Co–CeO_2_ amount on the catalytic reduction of MO was assessed (Figure 5). Two, four, and six beads of Cu@Alg/Co–CeO_2_ were added individually to the MO solutions having the same volume (2.5 mL) and concentration (0.07 mM) of MO, as well as volume (0.25 mL) and concentration (0.1 M) of NaBH_4_. Figure 5d demonstrates the % reduction vs. time plot for the present study. Initially, various numbers of Cu@Alg/Co–CeO_2_ beads, i.e., two, four, and six beads, were utilized and tested as catalysts for MO reduction using NaBH_4_. The recorded absorbance by UV–vis evidently showed a continuous decrease in absorbance with the passage of time, and this decrease in absorbance was much faster in the case of six beads as compared to four beads and two beads, i.e., increasing the catalyst amount increased the rate of reduction. Different amounts of Cu@Alg/Co–CeO_2_ decolorized MO solutions in 9.0, 9.0, and 32.0 min, respectively. Hence, it was discovered that a high amount of Cu@Alg/Co–CeO_2_ helps to reduce MO faster as compared to a smaller amount, and thus six beads of Cu@Alg/Co–CeO_2_ were more effective than four and two beads. The examined solutions containing 0.07 mM MO were completely decolorized within 9.0 min using six beads of Cu@Alg/Co–CeO_2_. However, decreasing the Cu@Alg/Co–CeO_2_ amount to two beads increased the time required for terminating the reduction to 32.0 min to completely reduce the MO. Figure 5d shows that increasing the Cu@Alg/Co–CeO_2_ amount from two beads to six beads caused a decrease in time for complete reduction of MO. The results indicated that a high amount of Cu@Alg/Co–CeO_2_ can eliminate MO from water more easily, owing to the exposure of more sites for the reduction of MO, and thus can quickly reduce MO as compared to a low quantity of Cu@Alg/Co–CeO_2_, which takes a long time to complete the reaction. Thus, increasing the beads’ number accelerates the reaction and reduces the time for reaction completion. The reason for the acceleration of the reduction reaction is that a high amount of Cu@Alg/Co–CeO_2_ beads offers a large surface area for the adsorption of reactants and desorption of products.

**Figure 6:**
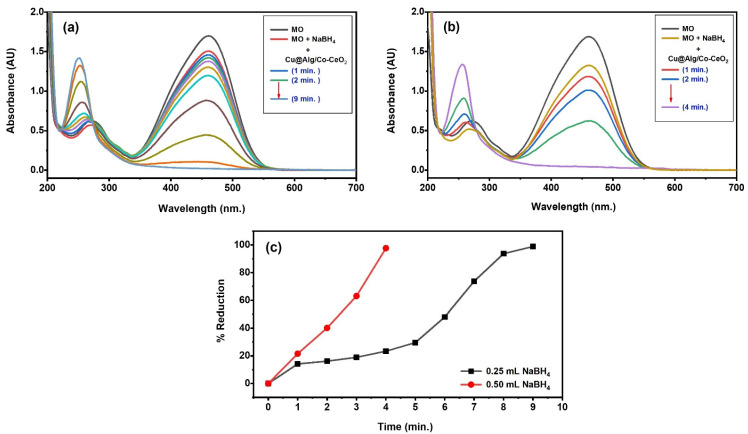
UV–vis absorption spectra for the reduction of MO (0.07 mM) using (**a**) 0.25 mL, (**b**) 0.5 mL NaBH_4_ (0.1 M), and Cu@Alg/Co–CeO_2_ catalyst (6 beads). (**c**) % Reduction of MO as a function of time.

The influence of NaBH_4_ amount (0.25 mL and 0.5 mL) was studied on the catalytic reduction of MO using six beads of Cu@Alg/Co–CeO_2_. As displayed in Figure 6, the decrease in NaBH_4_ concentration led to more time for completing the reduction process. This is ascribed to the significant role of the reducing agent and catalyst since treatment of pollutants with NaBH_4_ alone cannot reduce time without a catalyst. MO was reduced up to more than 97% with NaBH_4_ (0.25 mL and 0.5 mL of 0.1 M) in 9.0 min and 4.0 min, respectively. Thus, we can conclude that the reduction rate becomes speedier with increasing the amount of NaBH_4_.

**Figure 7:**
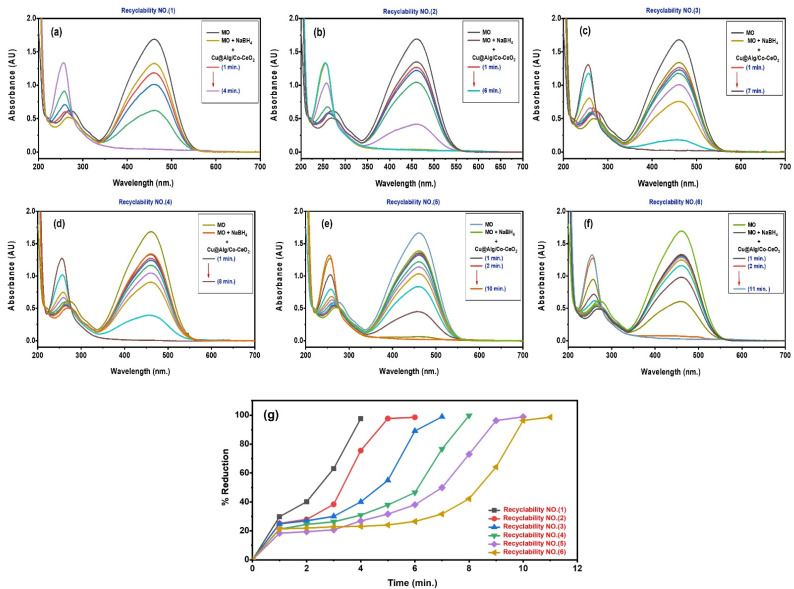
UV–vis absorption spectra for the reduction of MO (0.07 mM) using 0.5 mL NaBH_4_ (0.1 M) and Cu@Alg/Co–CeO_2_ catalyst (6 beads): (**a**) 1st cycle, (**b**) 2nd cycle, (**c**) 3rd cycle, (**d**) 4th cycle, (**e**) 5th cycle, and (**f**) 6th cycle. (**g**) % Reduction of MO as a function of time.

The reusability of Cu@Alg/Co–CeO_2_ was assessed for MO reduction. Stability and recyclability were clearly observed several times with a slight loss in catalytic activity. Figure 7 represents the time taken for MO reduction in each cycle using the same Cu@Alg/Co–CeO_2_ beads, which means sensible recyclability of Cu@Alg/Co–CeO_2_. The recyclability results signify the stability and recyclability of Cu@Alg/Co–CeO_2_, which reduced MO in 12 min until the 6th cycle. The results exhibited that the required time for completing the reaction increased within six consecutive reusability times. These data indicate that Cu@Alg/Co–CeO_2_ has plausible reusability and stability. Figure 7g reveals that Cu@Alg/Co–CeO_2_ reduced MO in 12 min even until the 6th cycle, signifying the efficient activity of Cu@Alg/Co–CeO_2_ toward MO reduction despite the slight loss in activity after each cycle, which is commonly observed in the majority of catalytic reactions, even with highly stable catalysts. The decrease in activity of Cu@Alg/Co–CeO_2_ could either be due to the oxidation or release of Cu nanoparticles from the support. Thus, the designed beads are efficient, stable, and recyclable, offering easy recovery from the reaction media. Thus, nanocomposition contributed a significant role in enhancing catalytic characteristics [42,51].

The possible mechanism for MO reduction in the presence of Cu@Alg/Co–CeO_2_ is shown in Scheme 1. Initially, BH_4_^−^ attacks MO molecules adsorbed on Cu@Alg/Co–CeO_2_, where transfer of electron and hydrogen takes place from BH_4_^−^ to MO. These electrons, which are carried via Cu@Alg/Co–CeO_2_, cause activation and breakage of azo bonds in MO [25] by converting –N=N– to –HN–NH– and then break down –HN–NH– to amines. So, decolorized MO solution indicates catalytic reduction of MO. Thus, Cu@Alg/Co–CeO_2_ transfers electrons from BH_4_^−^ (donor) to MO (acceptor) and thus causes acceleration of the MO reduction process.

#### 3.2.2. Photocatalytic Degradation

**Figure 8:**
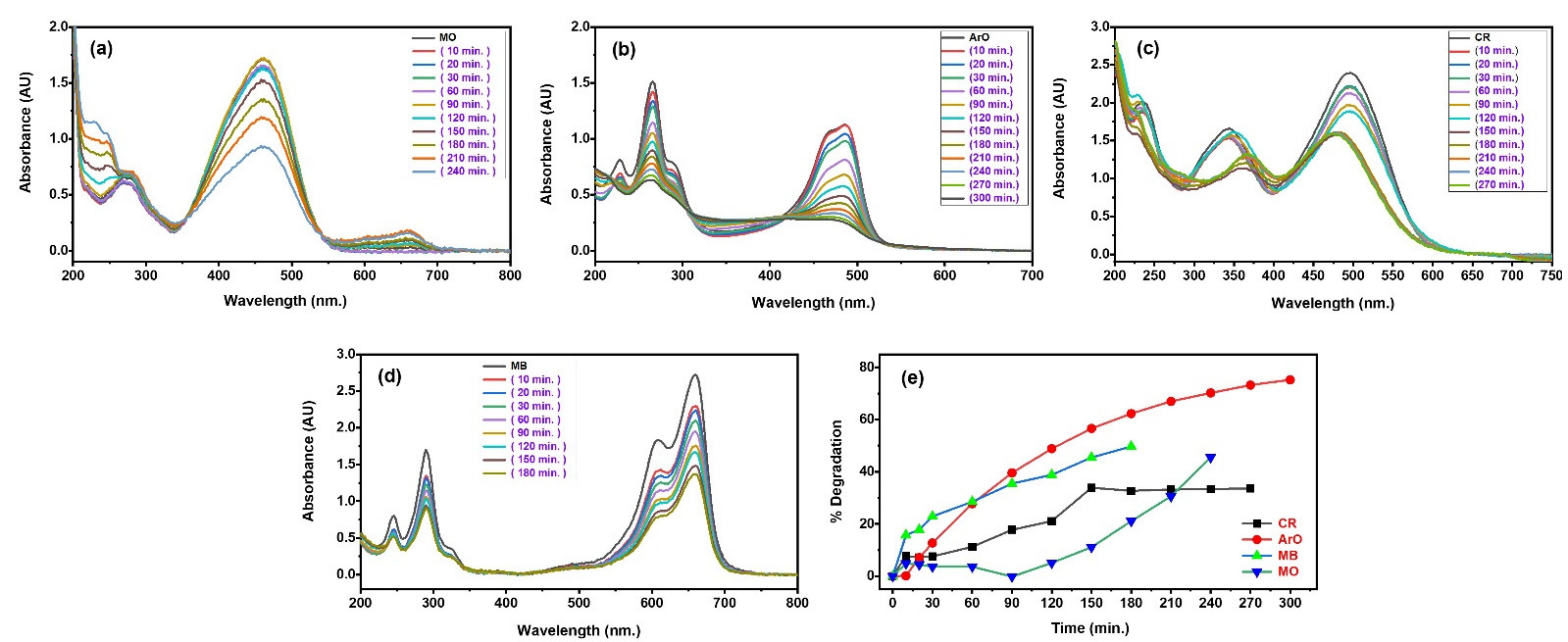
UV–vis absorption spectra for the photodegradation of (**a**) MO (0.07 mM), (**b**) ArO (0.07 mM), (**c**) CR (0.07 mM), and (**d**) MB (0.07 mM) under solar light using Cu@Alg/Co–CeO_2_ catalyst (4 beads). (**e**) % Degradation of ArO as a function of time.

In this study, the removal of dyes was carried out under solar light using Cu@Alg/Co–CeO_2_ as a photocatalyst instead of reducing agents. The effectiveness of Cu@Alg/Co–CeO_2_ beads was tested for the photocatalytic degradation of a series of pollutants such as MO (0.07 mM), ArO (0.07 mM), CR (0.07 mM), and MB (0.07 mM) under solar light. Catalytic degradation was performed using Cu@Alg/Co–CeO_2_ under solar light irridiation (Figure 8a–d). Similar experimental conditions were applied for all contaminants. The wavelengths were MO, 460 nm; ArO, 485 nm; CR, 495 nm; and MB, 659 nm. After the addition of Cu@Alg/Co–CeO_2_, a decrease in peak intensity was noticed with time, indicating the photodegradation of dyes by Cu@Alg/Co–CeO_2_ under solar light. Figure 8 displays that the absorbance band of dyes decreased steadily while completing the degradation reaction. Figure 8 indicates that degradation of ArO is faster among all studied dyes, which indicates that Cu@Alg/Co–CeO_2_ degraded ArO more efficiently. Cu@Alg/Co–CeO_2_ degraded ArO in 5 h, while Cu@Alg/Co–CeO_2_ did not degrade other dyes even in 5 h.

The % degradation was calculated by Equation (1), and the % degradation of different dyes is shown in Figure 8e, where degradation of ArO reached about 75.26% in 5 h, while low photodegradation was achieved for other dyes. The obtained data of Cu@Alg/Co–CeO_2_ activity in degrading dyes under solar light are as follows: MO, 45.54% at 240 min; ArO, 75.26% at 300 min; CR, 33.65% at 270 min; and MB, 49.70% at 180 min (Figure 8e). Cu@Alg/Co–CeO_2_ demonstrated superior catalytic activity toward ArO under sunlight. Thus, Cu@Alg/Co–CeO_2_ was more effective and efficient in the reduction of ArO. The fast decrease in absorption suggests that Cu@Alg/Co–CeO_2_ beads are effective catalysts for the degradation and mutagenic disruption of ArO, thus boosting photocatalytic activity. This study showed that Cu@Alg/Co–CeO_2_ beads are the better catalyst in the photodegradation of ArO dye. In addition, it was noticed that ArO was photodegraded by around 75% by Cu@Alg/Co–CeO_2_ among the tested dyes. This behavior suggests that Cu@Alg/Co–CeO_2_ is a perfect choice for ArO degradation. Therefore, ArO was chosen for detailed analysis, where we optimized the catalyst amount and recyclability.

**Figure 9:**
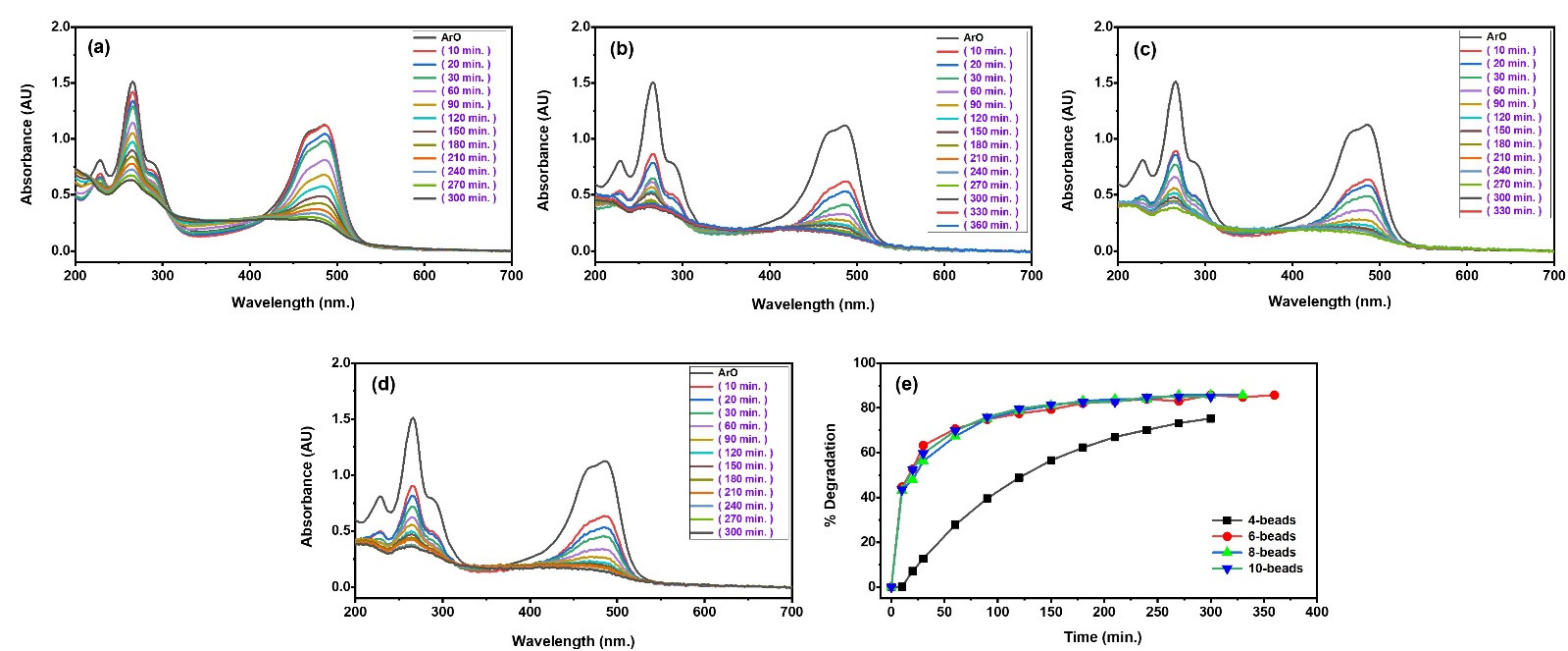
UV–vis absorption spectra for the photodegradation of ArO (0.07 mM) under solar light using (**a**) 4 beads, (**b**) 6 beads, (**c**) 8 beads, and (**d**) 10 beads of Cu@Alg/Co–CeO_2_ catalyst. (**e**) % Degradation of ArO as a function of time.

As the catalyst amount plays a significant role in photocatalysis, we studied the degradation of ArO using different catalyst dosages. The effect of catalyst amount on reaction rate was investigated by adding four different amounts (4 beads, 6 beads, 8 beads, and 10 beads) of Cu@Alg/Co–CeO_2_ for the degradation of 0.07 mM ArO under solar light conditions (Figure 9). Figure 9e displays % degradation vs. time. It is clear that degradation rate increases with increasing catalyst amount. ArO concentration decreased very fast in the case of 10 beads, and thus the effect of Cu@Alg/Co–CeO_2_ quantity led to a positive impact on photodegradation by increasing the total number of active sites. These findings indicate that the photocatalytic reaction is related to the Cu@Alg/Co–CeO_2_ quantity, i.e., the photocatalytic degradation increases with an increasing amount of Cu@Alg/Co–CeO_2_. A decrease in ArO absorbance was noticed with increasing time, indicating high removal percentage for ArO using 10 beads of Cu@Alg/Co–CeO_2_ under solar light, which indicates that Cu@Alg/Co–CeO_2_ amount plays an important role in ArO photodegradation. Figure 9 presents that 85% of ArO was degraded in 5 h using 10 beads, while % degradation for ArO was 75.27% (4 beads), 85.71% (6 beads), and 85.71% (8 beads).

**Figure 10:**
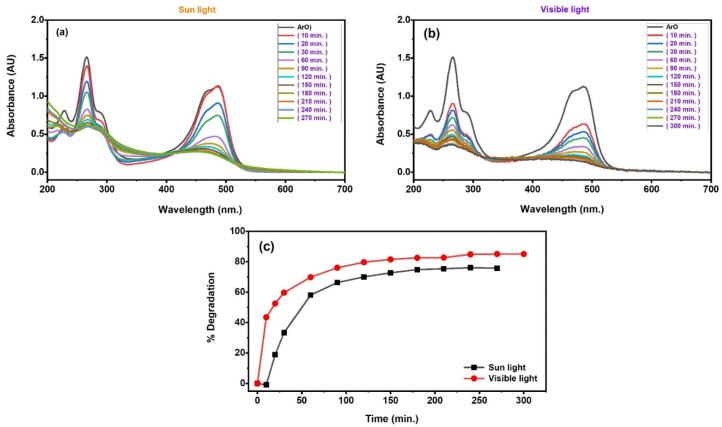
UV–vis absorption spectra for the photodegradation of ArO (0.07 mM) under (**a**) sunlight and (**b**) solar light using Cu@Alg/Co–CeO_2_ catalyst (10 beads). (**c**) % Degradation of ArO as a function of time.

Light plays a significant role in photocatalysis, so we determined the degradation of ArO under sunlight compared with solar light produced by a solar simulator. Figure 10 represents the effect of light, where degradation under the solar simulator light is slightly high as compared to sunlight. Solar produces pure solar light, while sunlight is a mixture of solar and UV light, which might be the reason for slightly lower degradation under sunlight. Figure 10 clearly indicates that the trend of photodegradation is similar; however, 75% of ArO was degraded under sunlight, while 85% of ArO was degraded under solar light using 10 beads.

**Figure 11:**
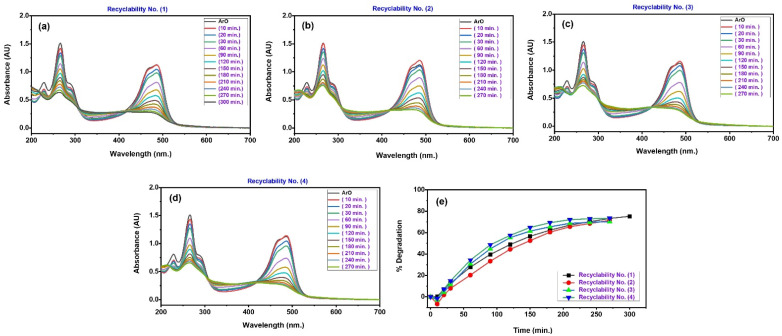
UV–vis absorption spectra for the photodegradation of ArO (0.07 mM) under solar light using Cu@Alg/Co–CeO_2_ catalyst (4 beads): (**a**) 1st cycle, (**b**) 2nd cycle, (**c**) 3rd cycle, and (**d**) 4th cycle. (**e**) % Degradation of ArO as a function of time.

Moreover, we studied the recyclability to explore the outstanding characteristics of Cu@Alg/Co–CeO_2_ in reusability for several cycles. First, four beads of Cu@Alg/Co–CeO_2_ were utilized, separated, washed with distilled water, dried, and reused in another run. Cu@Alg/Co–CeO_2_ was reused for four cycles. Figure 11 reveals degradation of ArO in the first cycle (% degradation = 75.27%), second cycle (% degradation = 71.44%), third cycle (% degradation = 70.19%), and fourth cycle (% degradation = 73.39%) at 270 min. It can clearly be observed that % degradation of ArO was nearly constant for all cycles, with a slight decrease in the reaction rate. Comparable results in the literature support the fact of obtaining a slight decrease in reaction rate by employing nanocatalysts in many cycles.

The photodegradation mechanism toward organic pollutants is described in Scheme 1. Photon absorption initially occurs due to Cu@Alg/Co–CeO_2_, which causes electron excitation from the valence band to the conduction band and generates a positive hole in the valence band:

$$\mathrm{Cu}@\mathrm{Alg}/\mathrm{Co}-{\mathrm{CeO}}_{2}+h\nu \to e_{CB}^{-}+h_{VB}^{+}\;$$

The excited electron in the conduction band ionizes the oxygen that is physically adsorbed on the surface of Cu@Alg/Co–CeO_2_.

$${\left(O_{2}\right)}_{ads}+e_{CB}^{-}\to O_{2}^{•-}$$

The positive holes convert the hydroxyl group (OH^−^) to ${\mathrm{OH}}^{•}$ and $O_{2}^{-•}$ to ${\mathrm{HO}}_{2}^{•}$.

$$(H_{2}O↔{\mathrm{OH}}^{-}+H^{+})+h_{VB}^{+}\to {\mathrm{OH}}^{•}+H^{+}$$

$$H^{+}+O_{2}^{-•}\to {\mathrm{HO}}_{2}^{•}$$

Moreover, ${\mathrm{HO}}_{2}^{•}$ is converted into O_2_ and H_2_O_2_.

$$2{\mathrm{HO}}_{2}^{•}\to O_{2}+H_{2}O_{2}$$

Further, H_2_O_2_ is decomposed to hydroxyl ion and hydroxyl radical.

$$H_{2}O_{2}\to {\mathrm{OH}}^{-}+{\mathrm{OH}}^{•}$$

Hydroxyl radical and positive holes cause oxidation of ArO.

$${\mathrm{OH}}^{•}+R\to \mathrm{OH}+R^{•}\;$$

$$R+h_{VB}^{+}\to R^{+•}\to degradation\;products\;$$

## 4. Conclusions

A facile synthesis of Cu@Alg/Co–CeO_2_ nanoparticles was performed, followed by an investigation of catalytic reduction and photocatalytic degradation of different dyes and nitrophenols. The results revealed that Cu@Alg/Co–CeO_2_ is an efficient catalyst that can effectively reduce and degrade MO and ArO in a short time with the aid of NaBH_4_ and solar light. Cu@Alg/Co–CeO_2_ can be simply eliminated from the reaction matrix, making it a recyclable and reusable catalyst. Cu@Alg/Co–CeO_2_ offered superb performance toward the catalytic reduction and photocatalytic degradation of MO and ArO, and thus the designed nanocatalyst possesses exceptional effectiveness for complete removal of MO and ArO, which can be promising for water purification purposes.

## Figures and Tables

**Table 1:** Characteristics of catalytic reduction of studied pollutants using NaBH_4_ and four beads of Cu@Alg/Co–CeO_2_.

Pollutant	% Reduction	Time (min)	Rate Constant (min^−1^)
4-NP	95.41	25	0.1267
ArO	47.76	30	0.0212
CR	93.27	26	0.1163
MB	99.73	13	0.2296
MO	97.51	11	0.3129
2,6-DNP	1.71	14	0.0008
2-NP	37.94	27	0.0188
K_3_[Fe(CN)_6_]	97.38	12	0.2615

## Data Availability

Not applicable.
